# Publisher Correction: Long-term functional and structural preservation of precision-cut human myocardium under continuous electromechanical stimulation in vitro

**DOI:** 10.1038/s41467-019-08510-9

**Published:** 2019-01-28

**Authors:** Carola Fischer, Hendrik Milting, Evelyn Fein, Elisabeth Reiser, Kun Lu, Thomas Seidel, Camilla Schinner, Thomas Schwarzmayr, Rene Schramm, Roland Tomasi, Britta Husse, Xiaochun Cao-Ehlker, Ulrich Pohl, Andreas Dendorfer

**Affiliations:** 1 Walter-Brendel-Centre of Experimental Medicine, University Hospital, LMU Munich, Marchioninistr. 27, 81377 Munich, Germany; 2 Erich & Hanna Klessmann Institute, Clinic for Thoracic and Cardiovascular Surgery, Heart and Diabetes Center NRW, Ruhr-University Bochum, Georgstr. 11, 32545 Bad Oeynhausen, Germany; 3 Institute of Cellular and Molecular Physiology, Friedrich-Alexander University Erlangen-Nürnberg, Waldstr. 8, 91054 Erlangen, Germany; 4 Institute of Anatomy, Ludwig-Maximilians-University Munich, Pettenkoferstr. 11, 80336 Munich,, Germany; 5 Institute of Human Genetics, Helmholtz Zentrum München, Ingolstädter Landstr. 1, 85764 Neuherberg, Germany; 6 Clinic of Cardiac Surgery, University Hospital, LMU Munich, Marchioninistr. 15, 81377 Munich, Germany; 7 Clinic of Anaesthesiology, University Hospital, LMU Munich, Marchioninistr. 15, 81377 Munich, Germany; 8 German Center for Cardiovascular Research, partner site Munich Heart Alliance, 80802 Munich, Germany

Correction to: *Nature Communications*; 10.1038/s41467-018-08003-1, published online 10 January 2019

The original version of this Article incorrectly acknowledged Elisabeth Reiser and Rene Schramm as a corresponding author. This has now been corrected in both the PDF and HTML versions of the Article.

