# Supplementary material for: Long-term functional and structural preservation of precision-cut human myocardium under continuous electromechanical stimulation in vitro
Source: Nat Commun. 2019 Jan 10;10:117. doi: 10.1038/s41467-018-08003-1 (PMC6328583; doi:10.1038/s41467-018-08003-1)
Supplement: Supplementary file 1 — Supplementary Information [file 41467_2018_8003_MOESM1_ESM.pdf]

**Supplementary information**

# Long-term functional and structural preservation of precision-cut human myocardium under continuous electromechanical stimulation *in vitro*

**Fischer *et al.***

## Supplementary Figures

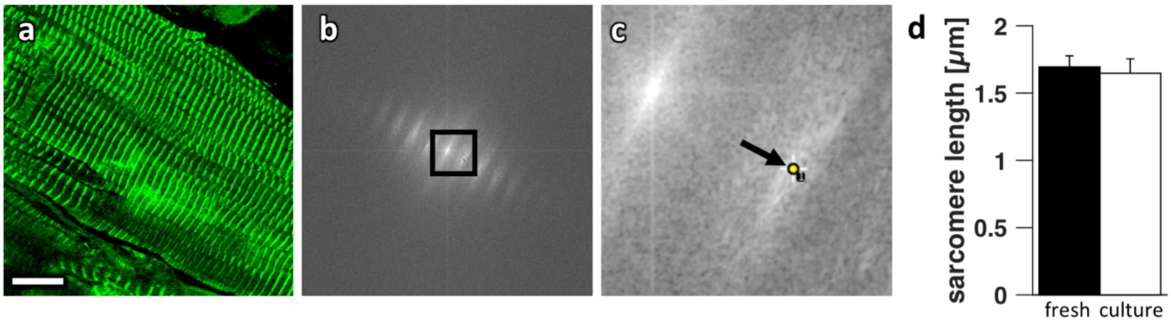


**Supplementary Figure 1** Sarcomere length in freshly cut and cultivated failing cardiac human tissue slices fixed without preload. **a** Confocal microscopic image of tissue stained for α-actinin, showing a regular z-disk pattern. Scale bar: 10µm. **b** Power spectrum of the Fourier transform of the image in *a*. **c** Zoom-in of boxed region in *b*. Arrow indicates the detected maximum of the power spectrum within the frequency range of 1 to 1/3 $\mu m^{-1}$, corresponding to a sarcomere length of 1.6 µm in the chosen example. **d** Mean sarcomere length calculated from three fresh and three paired cultured tissue slices. Sarcomere length per sample was obtained by analyzing 3-6 images per tissue slice. Error bars represent SEM.

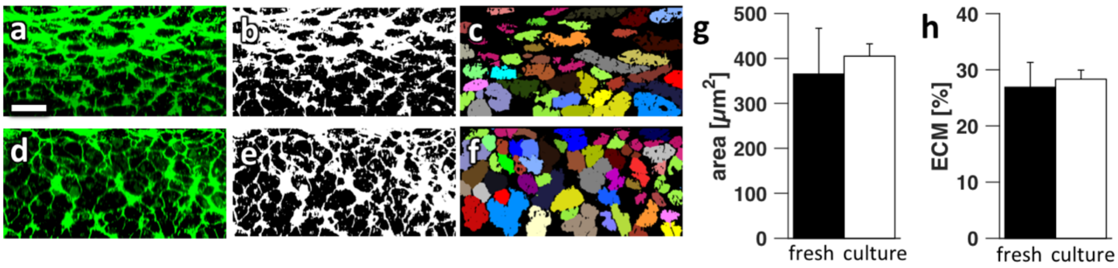


**Supplementary Figure 2** Myocyte cross section area in freshly cut and cultured failing cardiac human tissue slices. **a-c** example of fresh tissue, **d-f** example of tissue cultured for 7 weeks. **a, d** Thick sections were stained with wheat germ agglutinin (WGA) and imaged with 3D confocal microscopy. **b, e** WGA signal was segmented by applying a histogram-based threshold of mode+1SD. **c, f** Semi-automated segmentation based on morphological watershed algorithms was used to identify cross sections of individual myocytes. **g** Mean cardiomyocyte cross section area obtained from three fresh and three paired cultured tissue slices. Cross section area per sample was obtained by analyzing 9 cells per tissue slice. Only cells completely in the stack were considered, and cross sections were measured near cell centers, where myocyte width was largest. **h** Percentage of extracellular matrix (ECM) as an indicator of fibrosis, derived from the volume fraction of the segmented WGA signal of four tissue slices and 7 image stacks per group. Error bars represent SEM. Scale bar: 20µm, applies to *a-f*.

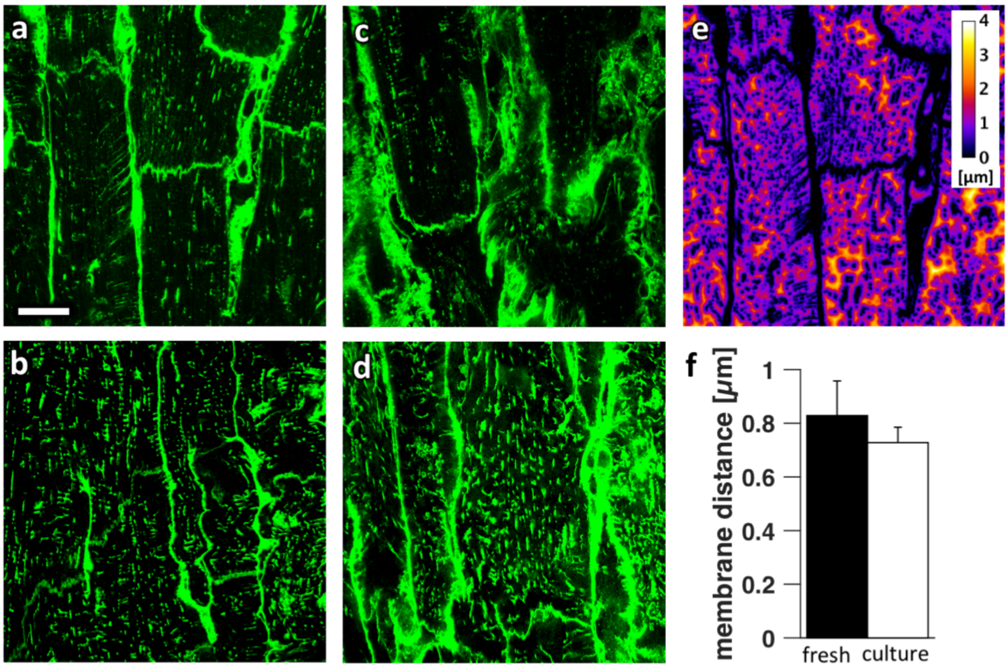


**Supplementary Figure 3** Transverse tubular system in freshly cut and cultured failing cardiac human tissue slices. Wheat germ agglutinin staining of **a-b** fresh tissue, **c-d** slices cultured for 7 weeks from paired samples, which did not show t-system depletion or changes in t-system geometry. **e** Distance transform of the image shown in *a*, indicating the distance to the nearest membrane at each pixel. **f** Mean membrane distance as a marker of t-system density did not show significant difference between fresh and cultured slices. Membrane distance was analyzed in three fresh and three paired cultured tissue slices, with 2-3 three-dimensional image stacks per tissue slice. Error bars represent SEM. Scale bar: 20µm, applies to *a-e*.

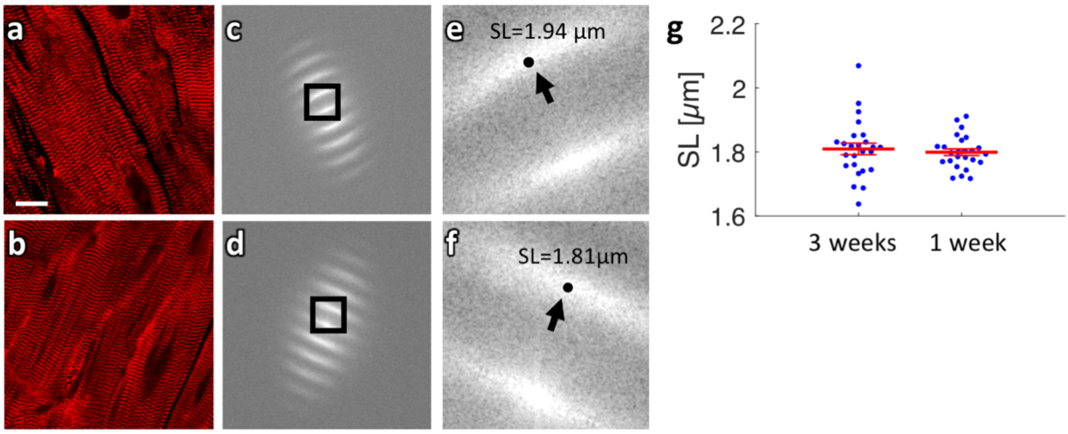


**Supplementary Figure 4** Sarcomere length (SL) in slices fixed under standard culture conditions. Two slices of failing myocardium obtained from different patients were fixed under continued stretch in the BMCCs after **a, c, e** cultivation for 3 weeks, or **b, d, f** cultivation for 1 week. **a-b** Confocal microscopic images of tissues stained for $\alpha$-actinin. Scale bars: 10µm. **c-d** Power spectra of the Fourier transform of the images in *a* and *b*, respectively. **e, f** Zoom-ins of boxed regions in *c* and *d*, respectively. Arrows indicate the detected maximum of the power spectrum within 1 to 1/3 $\mu m^{-1}$. **g** Distribution, mean and SEM of sarcomere length in 25 analyzed areas of each sample. Mean values of both tissues were identical at 1.8 µm. Scale bar: 10 µm, applies to *a-b.*

## Supplementary Tables

**Supplementary Table 1** Patient characteristics

| age | sex | heart disease | myocardial infarction | CHD | PAH | atrial fibrillation | inflammation | valvular defect | ICD | LVAD | other diagnoses | EF (%) | LVEDD (mm) | septal thickness (mm) | familial disposition | gene mutation identied | tissue transport | stability in culture |
|---|---|---|---|---|---|---|---|---|---|---|---|---|---|---|---|---|---|---|
| 56 | m | DCM | | X | | | X | X | X | | sarcoidosis | 25 | 55 | 12 | | | X | +++ |
| 63 | m | DCM | | X | | | X | | X | | | 31 | 64 | 11 | X | X | X | +++ |
| 40 | m | DCM | | X | X | | | X | X | | | 20 | 59 | 8 | | | X | +++ |
| 50 | m | DCM | | | X | X | | X | X | | | 15 | 57 | 11 | | X | X | +++ |
| 44 | f | DCM | | | | | | | X | | hypothyreodism | | 61 | | X | X | | +++ |
| 56 | m | DCM | | | X | | X | | | X | sarcoidosis | 23 | 71 | | | | | +++ |
| 51 | m | DCM | | | | | | | X | | non compaction | 26 | 65 | | X | | | +++ |
| 65 | m | DCM | | | X | | | | X | | | 26 | 60 | | | | | +++ |
| 48 | m | DCM | | | | | | | X | | | 15 | 64 | | | | | +++ |
| 58 | m | DCM | | | X | | | X | X | X | | 20 | 47 | 11 | | | X | +++ |
| 63 | m | DCM | | | X | X | | X | X | | renal failure | 28 | 66 | 10 | | | X | +++ |
| 60 | m | AOO | | | | | | | | | no heart failure | | | | | | | +++ |
| 43 | m | ICM | | X | X | | | X | | | hypertension | 25 | 74 | 10 | X | | X | ++ |
| 62 | m | HCM | | | | X | | | X | | | 36 | 53 | 12 | | | X | ++ |
| 34 | m | DCM | | X | X | | X | X | | | non compaction | 25 | 71 | 10 | X | X | X | ++ |
| 63 | m | ICM | X | X | X | | | X | X | | diabetes II | 19 | 90 | 8 | | | X | + |
| 55 | m | ICM | X | X | | | | | | X | | 25 | 48 | 11 | | | X | + |
| 52 | m | DCM | | | | | | X | X | | | 26 | 78 | | | | | + |
| 48 | m | DCM | | X | | | X | | X | X | anemia | 29 | 46 | 11 | | | X | + |

Abbreviations: DCM: dilated cardiomyopathy, ICM: ischemic cardiomyopathy, HCM: hypertrophic cardiomyopathy (non obstructive), AOO: aortic outflow obstruction, CHD: coronary heart disease, PAH: pulmonary arterial hypertension, ICD: implantable cardioverter defibrillator, LVAD: left ventricular assist device, EF: ejection fraction, LVEDD: left ventricular enddiastolic diameter. Stability definitions: +++ contractility >4 weeks, ++ contractility <4 weeks, + contractility <2 weeks.

**Supplementary Table 2** Regulation of functional gene ontologies during myocardial culture.

| | Log2 of mRNA ratio to uncultured tissue | | | | | |
|---|---|---|---|---|---|---|
| Gene ontology | 8 days | 14 days | 24 days | 35 days | inter-cept | slope x35 |
| GO:0098735_positive regulation of the force of heart contraction | -1.55 | -1.06 | -1.25 | -0.32 | -1.82 | 1.34 |
| GO:0001985_negative regulation of heart rate involved in baroreceptor response to increased systemic arterial blood pressure | -0.37 | 1.96 | 0.69 | 2.33 | -0.26 | 2.44 |
| GO:0003059_positive regulation of the force of heart contraction by epinephrine | -0.73 | -0.61 | -1.07 | -0.66 | -0.71 | -0.09 |
| GO:0003065_positive regulation of heart rate by epinephrine | -1.48 | -2.46 | -1.37 | -1.85 | -1.84 | 0.09 |
| GO:1901899_positive regulation of relaxation of cardiac muscle | -2.57 | -1.21 | -1.46 | -0.76 | -2.58 | 1.86 |
| GO:0086006_voltage-gated sodium channel activity involved in cardiac muscle cell action potential | -1.06 | 0.12 | -0.65 | 0.17 | -0.95 | 1.03 |
| GO:0086008_voltage-gated potassium channel activity involved in cardiac muscle cell action potential repolarization | -1.08 | -0.70 | -0.41 | -0.39 | -1.14 | 0.85 |
| GO:0010881_regulation of cardiac muscle contraction by regulation of the release of sequestered calcium ion | -1.03 | -0.83 | -0.85 | -0.56 | -1.13 | 0.53 |
| GO:0086007_voltage-gated calcium channel activity involved in cardiac muscle cell action potential | -0.62 | 1.24 | -0.19 | 2.24 | -0.87 | 2.66 |
| GO:0086008_voltage-gated potassium channel activity involved in cardiac muscle cell action potential repolarization | -1.08 | -0.70 | -0.41 | -0.39 | -1.14 | 0.85 |
| GO:0086012_membrane depolarization during cardiac muscle cell action potential | -1.40 | -0.75 | -0.90 | -0.45 | -1.44 | 0.97 |
| GO:0071691_cardiac muscle thin filament assembly | -1.63 | -0.94 | -0.45 | -0.29 | -1.79 | 1.66 |
| GO:0035755_cardiolipin hydrolase activity | -1.89 | -1.31 | -0.70 | -0.23 | -2.25 | 2.11 |
| GO:0090082_positive regulation of heart induction by negative regulation of canonical Wnt signaling pathway | 1.50 | -0.12 | 1.09 | 0.09 | 1.23 | -1.01 |
| GO:0090271_positive regulation of fibroblast growth factor production | 0.63 | 1.19 | 0.06 | 0.24 | 1.08 | -0.95 |
| hsa00071 Fatty acid metabolism | -1.02 | -0.99 | -0.87 | -0.83 | -1.08 | 0.26 |
| hsa00640 Propanoate metabolism | -1.24 | -0.94 | -0.84 | -0.80 | -1.25 | 0.51 |
| hsa00010 Glycolysis / Gluconeogenesis | -0.52 | -0.15 | -0.27 | -0.32 | -0.39 | 0.13 |
| hsa00190 Oxidative phosphorylation | -0.81 | -1.37 | -0.90 | -1.32 | -0.89 | -0.36 |

Displayed data represent the expression of genes assembled in functional ontologies or biochemical pathways as logarithmic ratios of cultured and uncultured tissues. Temporal alteration of gene expression was approximated by linear regression that yielded estimations of expression levels at the start of cultivation (intercept), and of the temporal trend during 35 days of culture (slope×35).

**Supplementary Methods: Step-by-step sequence of heart slice preparation**

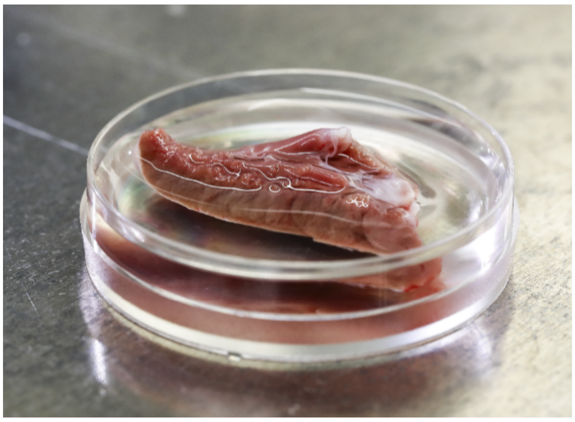

**a** Transmural section of left ventricular heart tissue is processed in cardioplegic HBSS on a cooled tray.

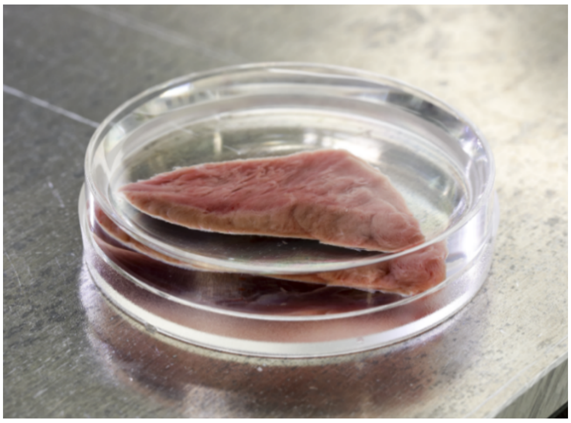

**b** Endocardial and trabecular layers are removed with scissors.

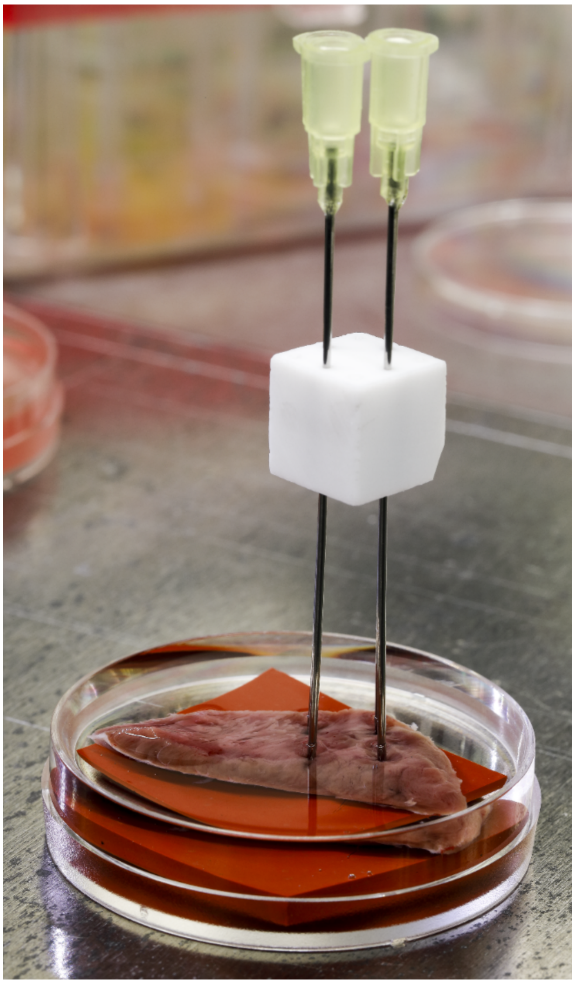

**c** Tissue is pinned down and a square area with best morphology (red color, no fibrosis, no retraction) is delimited by long puncture needles.

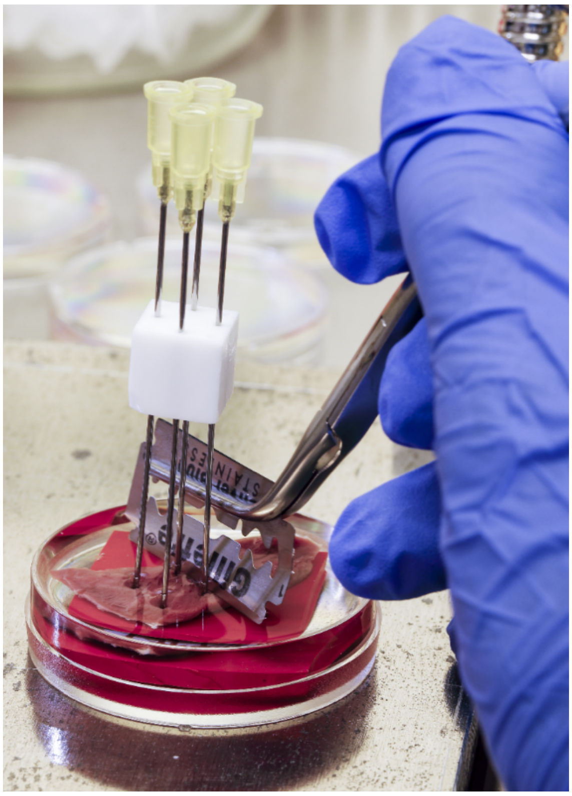

**d** Heart tissue is trimmed with a razor blade along the puncture needles resulting in a tissue block of about 8x8 mm².

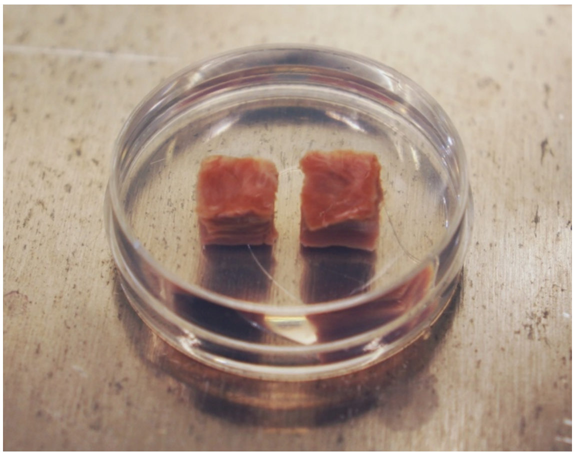

**e** Blocks of heart tissue are embedded in 4% low melt agarose with the epicardial surface facing down. Two blocks can be processed in parallel.

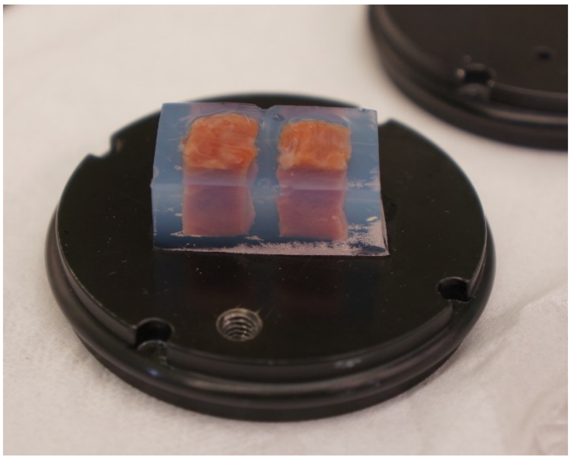

**f** After removal of excessive agarose, the block is attached with a drop (20 µL) of Histoacryl glue to the holder of the vibratome.

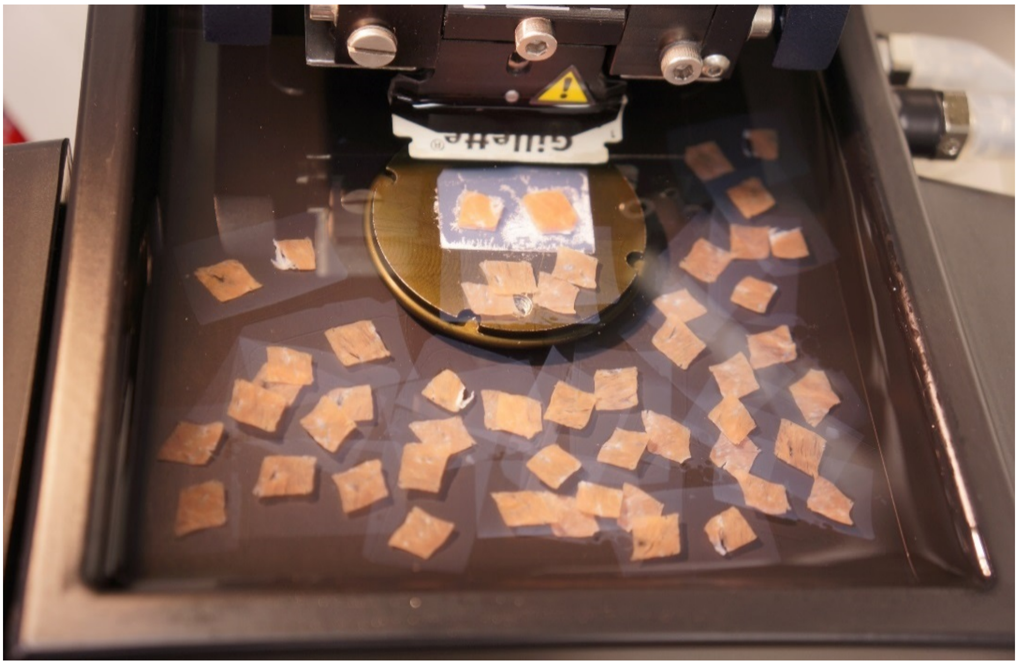

**g** Slicing is performed at 4 °C in the cooled bath of the vibratome. A vibrating razor blade (1.5 mm amplitude) is advanced at 0.07 mm $s^{-1}$ in layers of 300 µm thickness over the tissue. Slices should not be stored for longer than 1 h in cardioplegic solution.

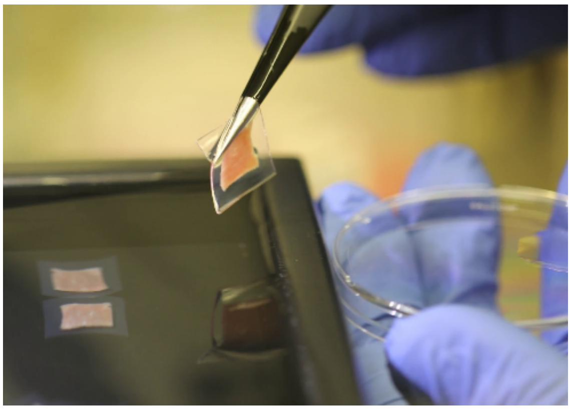

**h** One slice embedded in agarose is transferred from the vibratome bath to the lid of a Petri dish.

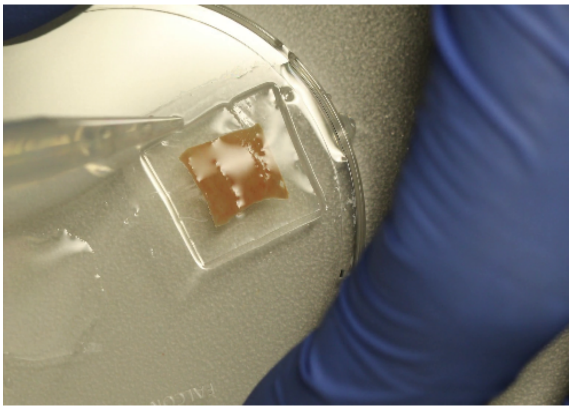

**i** Excess of slicing buffer is removed.

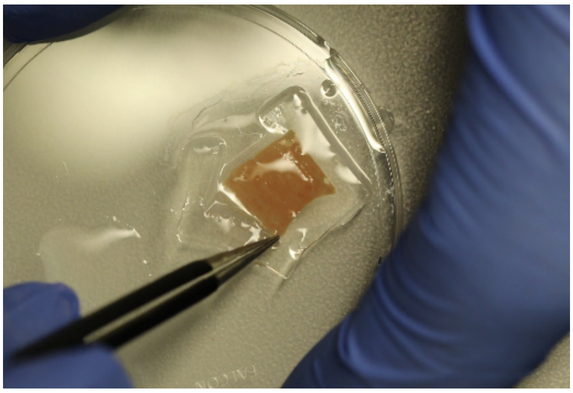

**j** Agarose is carefully detached and separated using tweezers.

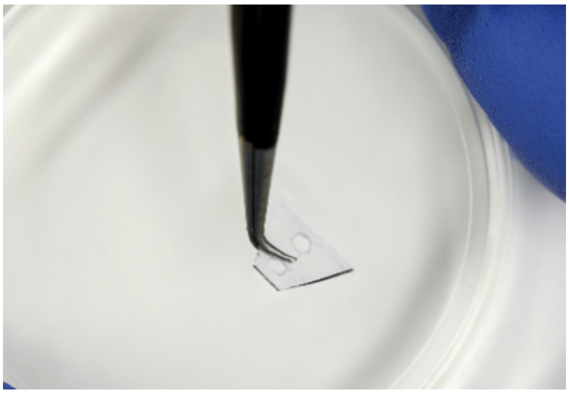

**k** 1 µL of Histoacryl glue is pipetted onto a Petri dish surface, and the wide edge of a plastic triangle is dipped into the layer.

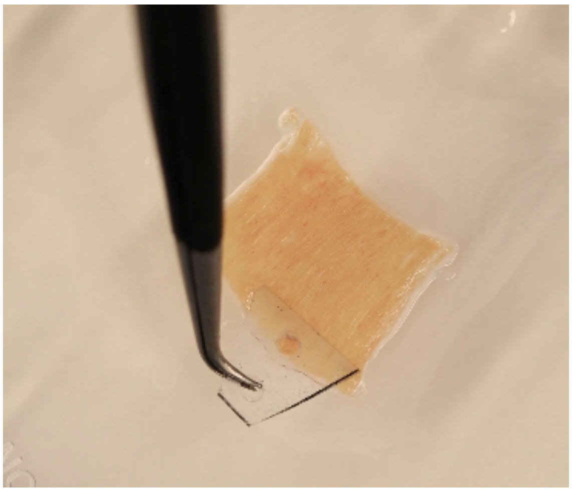

**l** Within 20 s, the first plastic triangle is carefully pressed onto one side of the heart slice with attention to rectangular orientation of the muscle fibres.

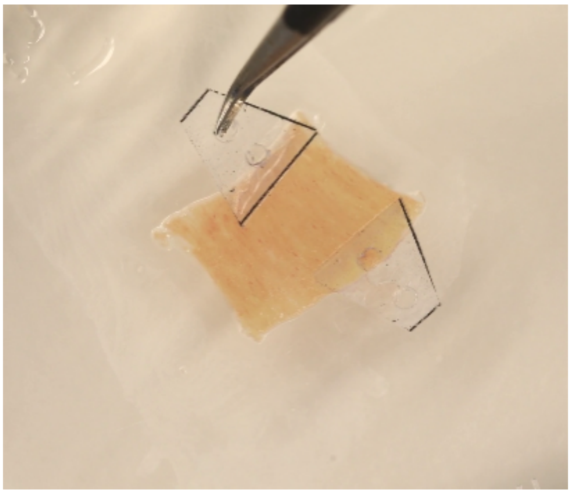

**m** The second plastic triangle is glued to the heart slice opposite to the first triangle.

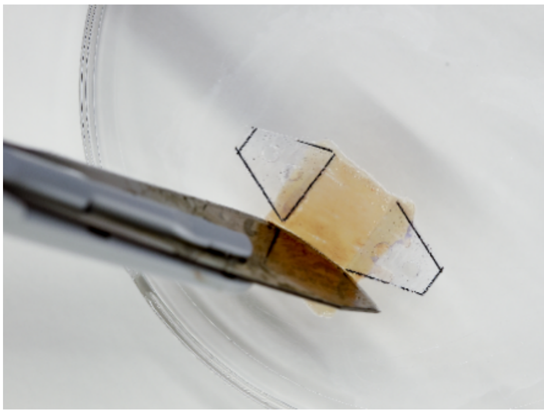

**n** The tissue is trimmed to the size specified by the triangle

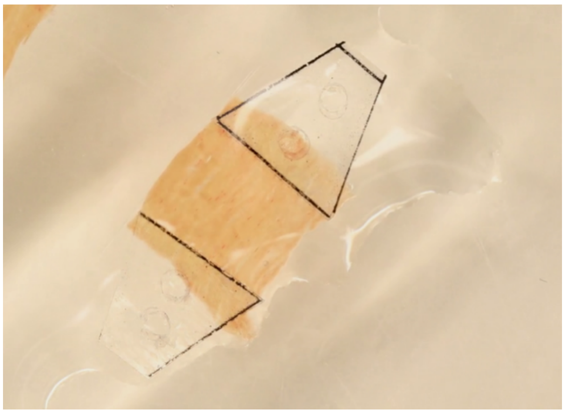

**o** Slice with attached plastic triangles can be stored in slicing buffer until mounting into a culture dish.

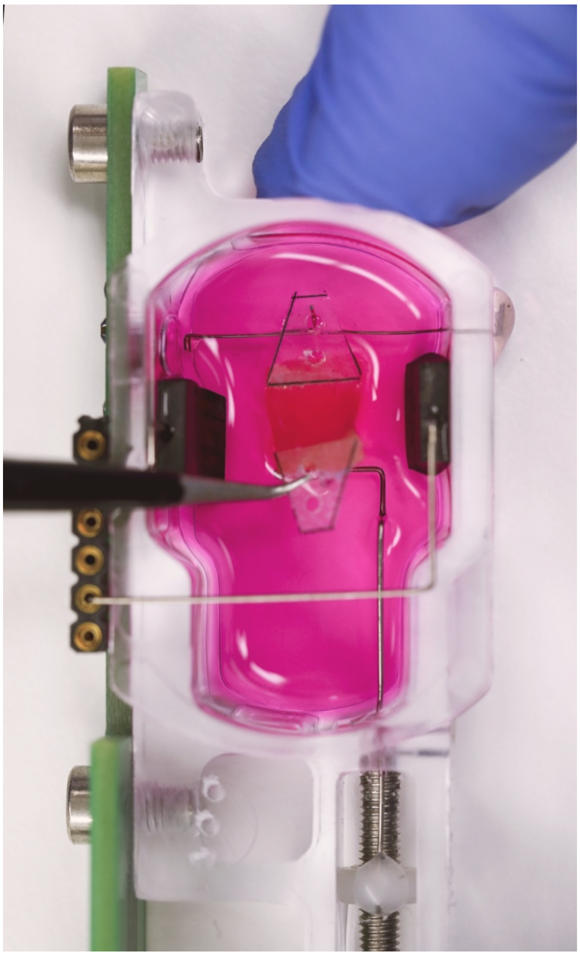

**p** Completely assembled culture dish with 2.4 mL medium has been equilibrated in the incubator. The slice is attached to the elastic spring wire by insertion of the wire pin into the hole of the (upper) triangle. The second triangle is hooked onto the movable wire which has been adjusted close to the elastic wire.

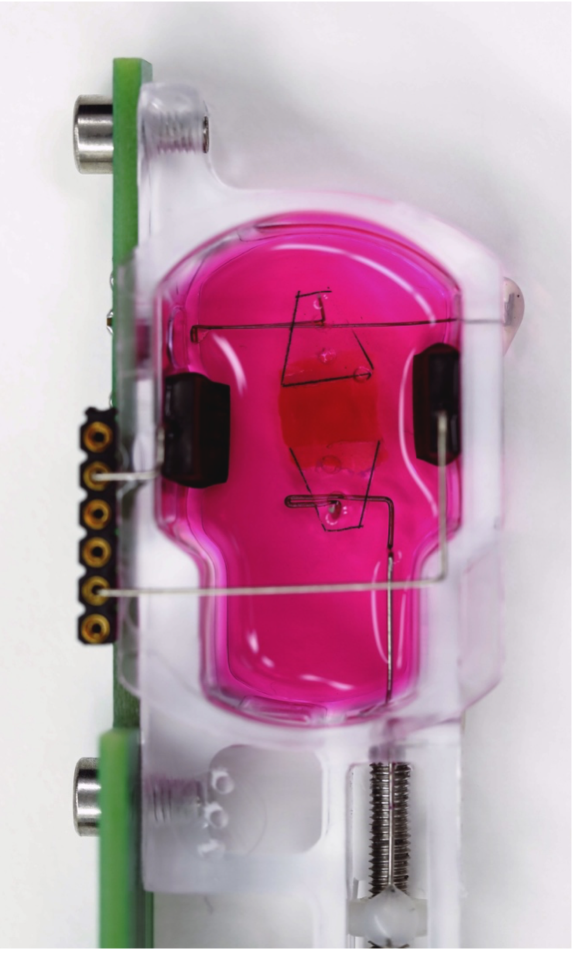

**q** The slice is submerged into the medium and stretched to slack length. After covering with a lid, the culture dish is transferred to the incubator. The dias-tolic force is then adjusted to a merely detectable level. After 1 h equilibration, the diastolic force is readjusted to 1 mN.
