# Supplementary material for: Long-term functional and structural preservation of precision-cut human myocardium under continuous electromechanical stimulation in vitro
Source: Nat Commun. 2019 Jan 10;10:117. doi: 10.1038/s41467-018-08003-1 (PMC6328583; doi:10.1038/s41467-018-08003-1)
Supplement: Supplementary file 3 — Description of Additional Supplementary Files [file 41467_2018_8003_MOESM3_ESM.docx]

**Description of Supplementary Files**

**File Name**: Supplementary Movie 1

**Description:** Demonstration of biomimetic culture assembly. The movie visualizes the process of holder attachment, tissue trimming, and mounting of myocardial slices into culture chambers.

**File Name:** Supplementary Data 1

**Description:** Full mRNA expression data underlying Table 1 and Supplementary Table 2. Copy numbers are given for each gene transcript identified by mRNA sequencing in myocardial tissues cultured for 8 - 35 days. Culture related alterations are expressed as Log2 values of the mRNA ratios of cultured vs. native tissues from corresponding samples.

**File Name:** Supplementary Data 2

**Description:** Source data of all results presented as averages of multiple experiments. Applies to Figs. 2a, 2d, 3a-d, 5b-c and Supplementary Figs. 1d, 2g-h, 3f, 4g.
