# Supplementary material for: Long-term functional and structural preservation of precision-cut human myocardium under continuous electromechanical stimulation in vitro
Source: Nat Commun. 2019 Jan 10;10:117. doi: 10.1038/s41467-018-08003-1 (PMC6328583; doi:10.1038/s41467-018-08003-1)
Supplement: Supplementary file 7 — Reporting Summary [file 41467_2018_8003_MOESM7_ESM.pdf]

nature research

Corresponding author(s): Andreas Dendorfer

Reporting Summary

Nature Research wishes to improve the reproducibility of the work that we publish. This form provides structure for consistency and transparency in reporting. For further information on Nature Research policies, see Authors & Referees and the Editorial Policy Checklist.

Statistical parameters

When statistical analyses are reported, confirm that the following items are present in the relevant location (e.g. figure legend, table legend, main text, or Methods section).

n/a	Confirmed	
☐	☒	The exact sample size (n) for each experimental group/condition, given as a discrete number and unit of measurement
☐	☒	An indication of whether measurements were taken from distinct samples or whether the same sample was measured repeatedly
☐	☒	The statistical test(s) used AND whether they are one- or two-sided<br>*Only common tests should be described solely by name; describe more complex techniques in the Methods section.*
☒	☐	A description of all covariates tested
☐	☒	A description of any assumptions or corrections, such as tests of normality and adjustment for multiple comparisons
☐	☒	A full description of the statistics including central tendency (e.g. means) or other basic estimates (e.g. regression coefficient) AND variation (e.g. standard deviation) or associated estimates of uncertainty (e.g. confidence intervals)
☐	☒	For null hypothesis testing, the test statistic (e.g. F, t, r) with confidence intervals, effect sizes, degrees of freedom and P value noted<br>*Give P values as exact values whenever suitable.*
☒	☐	For Bayesian analysis, information on the choice of priors and Markov chain Monte Carlo settings
☒	☐	For hierarchical and complex designs, identification of the appropriate level for tests and full reporting of outcomes
☒	☐	Estimates of effect sizes (e.g. Cohen's d, Pearson's r), indicating how they were calculated
☐	☒	Clearly defined error bars<br>*State explicitly what error bars represent (e.g. SD, SE, CI)*

Our web collection on statistics for biologists may be useful.

Software and code

Policy information about availability of computer code

Data collection	The programs LabChart 5, WinEDR 3.6 and Leica LAS X 3 were used for data recording and image acquisition. Firmware for operating the biomimetic culture setup was developed with Kinetis Design Studio 3. Control of tissue stimulation and data recording was performed by a custom-developed software as described in the "Methods" section. All custom-developed software will be made available to other investigators if requested.
Data analysis	Data were analyzed using LabChart Reader 7 or 8, and GraphPad Prism 5.

For manuscripts utilizing custom algorithms or software that are central to the research but not yet described in published literature, software must be made available to editors/reviewers upon request. We strongly encourage code deposition in a community repository (e.g. GitHub). See the Nature Research guidelines for submitting code & software for further information.

Data

Policy information about availability of data

All manuscripts must include a data availability statement. This statement should provide the following information, where applicable:

- Accession codes, unique identifiers, or web links for publicly available datasets
- A list of figures that have associated raw data
- A description of any restrictions on data availability

There are no restrictions in data availability. Source data of Figs. 2a, 2d, 3a-d, 5b-c and Supplementary Figs. 1d, 2g-h, 3f, 4g are provided as a Source Data file. Complete transcriptome data can be accessed under http://www.ncbi.nlm.nih.gov/bioproject/496588.

Field-specific reporting

Please select the best fit for your research. If you are not sure, read the appropriate sections before making your selection.

☒ Life sciences ☐ Behavioural & social sciences ☐ Ecological, evolutionary & environmental sciences

For a reference copy of the document with all sections, see nature.com/authors/policies/ReportingSummary-flat.pdf

Life sciences study design

All studies must disclose on these points even when the disclosure is negative.

Sample size	All tissue samples accessible during the study period (19 in total) were utilized for slice preparation and culture. Statistical methods could not be used to predetermine sample size since the variability of clinical samples could not be estimated prospectively. Instead, sample sizes were chosen with consideration of the variability and importance of each assay. Comparisons that included the variability of tissues from different patients (e.g. Figs. 2d, 3a-d) were reproduced with 6-15 biologically different tissues. Intraindividual treatment effects (e.g. Fig. 5bc) were tested in 5 samples. Descriptive observations were confirmed in at least 3 independent experiments.
Data exclusions	No data were excluded from the analyses.
Replication	All replications performed with the indicated sample sizes conformed with the conclusions of the study.
Randomization	Randomization was not feasible for this study because most of the experiments were determined by the availability of tissue samples and the time requirements of tissue culture.
Blinding	Blinding was not feasible for this study since culture conditions and experimental protocols had to be developed in parallel. Most of the data have been acquired by calibrated technical devices, and standardized analytical procedures were applied in order to avoid investigator bias.

Reporting for specific materials, systems and methods

Materials & experimental systems

n/a	Involved in the study	
☒	☐	Unique biological materials
☐	☒	Antibodies
☒	☐	Eukaryotic cell lines
☒	☐	Palaeontology
☒	☐	Animals and other organisms
☐	☒	Human research participants

Methods

n/a	Involved in the study	
☒	☐	ChIP-seq
☒	☐	Flow cytometry
☒	☐	MRI-based neuroimaging

Antibodies

Antibodies used	All antibodies and their sources are specifically described in the paragraphs "Histology" and "Morphmetric analysis of myocyte structure" within the "Methods" section.
Validation	All antibodies have been approved for immunostaining of human tissue, according to the specifications provided by the manufacturers. Only common antibodies with well-proven specificities were used. Staining of structures with the typical distribution of the specific targets in control tissues confirmed the suitability of the antibodies for the purpose of this study.

Human research participants

Policy information about studies involving human research participants

Population characteristics	All patients receiving a heart transplant at the two Clinics were involved in the study. In a single case, tissue was obtained from therapeutic resection of hypertrophic myocardium.
Recruitment	Patients were asked to participate in this study upon admission to the transplantation program, or during the consultation sessions preceding surgical interventions. Patients provided informed consent to the scientific use of their tissues.
